# Molecular cytogenetic characterization of chromosomal rearrangements - utility in genetic counseling and research

**DOI:** 10.1186/1755-8166-7-S1-I12

**Published:** 2014-01-21

**Authors:** Ashwin Dalal

**Affiliations:** 1 Diagnostics Division, Centre for DNA Fingerprinting and Diagnostics, Hyderabad, Andhra Pradesh, India

## 

Chromosomal abnormalities can lead to a number of human genetic disorders. A large number of chromosomal rearrangements are known to be associated with a disease phenotype as a result of interrupting or modifying the expression of gene(s) localized in or close to the breakpoints. Structural changes in chromosomes can be classified according to cytological types and their effect on the phenotype. The main structural rearrangements are translocations, inversions, deletions, insertions, isochromosomes, dicentric chromosomes and ring chromosomes. Structural rearrangements alter the genome architechture and may result in human disease phenotypes. The identification of genes involved in human diseases resulting from chromosomal rearrangements is important to understand the pathophysiology of the disease, further it often provides new insights into normal human development and biology. Chromosomal analysis with routine methods gives a low resolution of about 5 Mb. Newer techniques like Fluorescent *In Situ* Hybridization (FISH), array Comparative Genomic Hybridization (CGH) have increased the resolution exponentially. These recent advancements in molecular cytogenetic techniques have made it possible to characterize the structural chromosomal rearrangements to very high resolution. Some of these techniques and their application to characterization of chromosomal rearrangements will be discussed using different case scenarios.

